# Comparison metrics and power trade-offs for BCI motor decoding circuit design

**DOI:** 10.3389/fnhum.2025.1547074

**Published:** 2025-03-12

**Authors:** Joe Saad, Adrian Evans, Ilan Jaoui, Victor Roux-Sibillon, Emmanuel Hardy, Lorena Anghel

**Affiliations:** ^1^Université Grenoble Alpes, CEA, LIST, Grenoble, France; ^2^Université Grenoble Alpes, CEA, Leti, Grenoble, France; ^3^Université Grenoble Alpes, CEA, CNRS, Grenoble INP, IRIG-Spintec Laboratory, Grenoble, France

**Keywords:** brain-computer interfaces (BCIs), motor decoding, electroencephalography, electrocorticography, microelectrode array, feature extraction, low-power circuits, system-on-chip (SoC)

## Abstract

Brain signal decoders are increasingly being used in early clinical trials for rehabilitation and assistive applications such as motor control and speech decoding. As many Brain-Computer Interfaces (BCIs) need to be deployed in battery-powered or implantable devices, signal decoding must be performed using low-power circuits. This paper reviews existing hardware systems for BCIs, with a focus on motor decoding, to better understand the factors influencing the power and algorithmic performance of such systems. We propose metrics to compare the energy efficiency of a broad range of on-chip decoding systems covering Electroencephalography (EEG), Electrocorticography (ECoG), and Microelectrode Array (MEA) signals. Our analysis shows that achieving a given classification rate requires an Input Data Rate (IDR) that can be empirically estimated, a finding that is helpful for sizing new BCI systems. Counter-intuitively, our findings show a negative correlation between the power consumption per channel (PpC) and the Information Transfer Rate (ITR). This suggests that increasing the number of channels can simultaneously reduce the PpC through hardware sharing and increase the ITR by providing new input data. In fact, for EEG and ECoG decoding circuits, the power consumption is dominated by the complexity of signal processing. To better understand how to minimize this power consumption, we review the optimizations used in state-of-the-art decoding circuits.

## 1 Introduction

Advances in decoding algorithms have now made it possible to extract information from brain signals. Relevant information such as motor intentions (Chen et al., 2022; Benabid et al., 2019; Chamanzar et al., 2017; Hammer et al., 2013; Spüler et al., 2014), speech (Shaeri et al., 2024; Metzger et al., 2022; Herff et al., 2019), epileptic seizures (Guirgis et al., 2013; Yoo et al., 2013) and Parkinson tremor state (Shin et al., 2022) can be detected. The information can be used in closed-loop systems such as deep brain stimulation applications for epileptic seizures (Fleming et al., 2023; Kavoosi et al., 2022; Shin et al., 2022; Stanslaski et al., 2012; Sridhara et al., 2011; Chen et al., 2010), and essential tremor (Fraczek et al., 2021; Opri et al., 2020). Some early medical products already exist for these applications (Thenaisie et al., 2021; Jarosiewicz and Morrell, 2021).

In addition, the extracted information can be used in experimental BCI (BCI) applications such as controlling an exoskeleton (Benabid et al., 2019) or generating stimulation patterns for impaired patients after spinal cord injury (Lorach et al., 2023; Younessi Heravi et al., 2023; Greiner et al., 2021). The focus of this work is hardware systems for BCI applications, as these have been less explored although they have significant potential for addressing motor rehabilitation.

Methods for brain signal recording can range from fully-invasive to non-invasive. MEAs (MEAs), implanted directly into the brain tissue, offer a high spatial resolution as they are able to capture single-neuron signals (Musk and Neuralink, 2019; Maynard et al., 1997) while ECoG (ECoG) arrays, placed on the surface of the brain, measure signals averaged over thousands of neurons with limited invasiveness (Matsushita et al., 2018; Mestais et al., 2015). Non-invasive techniques can also be used for brain signal acquisition. EEG (EEG) signals detect electric signals averaged over a larger number of neurons than ECoG (Shokoueinejad et al., 2019) and can be used for brain signal decoding (Wu et al., 2024; Chamanzar et al., 2017; Wang et al., 2016; Sridhara et al., 2011).

Other non-invasive methods include MEG (MEG) and fNIRS (fNIRS). MEG measures magnetic activity in neurons, using machines that are physically large, hence not portable. The fNIRS method is based on detecting changes in hemodynamic activity by measuring variations in oxyhemoglobin and deoxyhemoglobin concentrations. Although fNIRS devices have become more portable, they do not meet the needs for real-time motor assistive applications, as they have a response-time of a few seconds (Ortega-Martinez et al., 2022), much slower than methods based on electrical signals.

General purpose microprocessors have a power consumption that is too high for battery-powered miniaturized medical applications. There is thus a growing need for custom hardware solutions that can decode brain signals using minimal power, while also meeting other well-known metrics such as accuracy and low-latency. The focus of this work is to analyze the state-of-the-art dedicated hardware platforms for BCI and to compare them using a set of metrics that we propose. The remainder of this paper is organized as follows. In Section 2, we present the articles that were included in this analysis. In Section 3, we propose metrics to analyze the performance of decoding circuits, then we compare the identified systems using these metrics. In Section 4, we highlight the most innovative power optimization techniques used in the selected circuits. In Section 5, we discuss the findings of our analysis and conclude with Section 6.

## 2 Literature review of BCI decoding circuits

### 2.1 Search methodology

We performed a review of the literature on hardware systems for brain signal decoding and identified papers presenting hardware systems that could be used for BCI motor decoding. The search was performed using PubMed, Scopus, Web of Science, IEEE Xplore and Google Scholar, and covered published work between 2010 and 2025, a period that witnessed a significant progress in chip development for BCI applications. Search queries were based on boolean combinations of BCI relevant keywords and expressions such as “Brain-computer interface,” “Motor decoding,” “Electroencephalography,"“Electrocorticography,” “Microelectrode Array” with others related to hardware such as “Hardware,” “Circuit,” “Chip,” “Low-power.” The queries were progressively refined to focus the results, and the search constraints were occasionally relaxed to explore a larger scope.

We mainly focused on circuits for BCI motor decoding, excluding systems that are meant to be used exclusively for neuromodulation or detection of epilepsy (Fleming et al., 2023; Tsai et al., 2023; Chua et al., 2022; O'Leary et al., 2018; Bin Altaf et al., 2015; Stanslaski et al., 2012) and tremor (Fraczek et al., 2021; Opri et al., 2020). We also did not include systems that only perform data acquisition (Lee et al., 2023; Reich et al., 2021; Lim et al., 2020; Matsushita et al., 2018; Mestais et al., 2015) or compression (Jang et al., 2023). In addition, although software approaches (Lorach et al., 2023; Chen et al., 2022; Yao et al., 2022; Volkova et al., 2019; Behrenbeck et al., 2019) are interesting for motor decoding, they have been excluded from the study as our aim is to compare BCI hardware systems. In general, circuits designed for SSVEP (SSVEP) decoding, such as Kartsch et al. (2019) have not been considered in the scope of the current study.

Although we focused on motor decoding, some systems with non-motor applications have been included. In fact, some emerging circuit techniques have potential to be used for future BCI motor applications. These exceptions are summarized as follows.

- The circuits presented in Sridhara et al. (2011) and Chen et al. (2010) have only been tested for epileptic seizure detection although they had been initially designed for general medical applications that require on-chip signal processing.
- The system in Shaeri et al. (2024) decodes text characters and is tested on auditory stimuli in mice. It was however included as we believe the innovative approach consisting of using an LDA (LDA) classifier with the DNC (DNC) features could be used for motor decoding.
- In Zhong et al. (2024), the authors describe a circuit that uses SSVEP to control a drone. Although we did not include circuits that exclusively decoded SSVEP, we consider this one an exception, as the decoding was used for a 4-DoF (DoF) control and the chip is one of a few systems that can perform online updates of the decoding model. Such method could be generalized for BCI motor applications.
- The circuit in Malekzadeh-Arasteh et al. (2020) describes an analog approach for feature extraction. Although it does not actually perform decoding, we have included it due its innovative approach. However, we only include it to discuss the power consumption and the optimizations that were applied. Similarly, the Neuralink circuit in Musk and Neuralink (2019) was also included in the power comparison. The systems in Liu et al. (2017) and Zhang et al. (2011) also extract analog features and Liu et al. (2017) implements a closed-loop neurostimulation. The main contributions of these two papers will be highlighted in Section 4.3.1 discussing emerging analog approaches.

### 2.2 State-of-the-art summary

**Figure 1:**
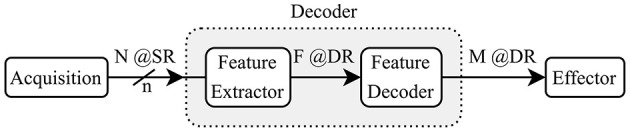
Reference model for a BCI Decoder showing a two-step decoding using feature extraction.

The block diagram in Figure 1 shows the main steps that a decoding system implements between the recorded brain signals and the outputs sent to the effector. The decoder takes in *N* channels, sampled at a given (*SR*) with a resolution of *n* bits and it outputs a classification as one of *M* classes with a (*DR*). For some decoders, an intermediate feature extraction step is applied before generating the outputs. In such cases, the decoder model can be split into two sub-modules: the feature extractor and the feature decoder. We assume that the features *F* are extracted at the same rate as the outputs (*DR*).

The reviewed works are listed in Table 1 where we show the type of signal that is decoded, the number of channels *N*, the input (*SR*), the bit resolution *n* of the ADC (ADC), the type of features, the extraction method, the number of features *F*, the type of task, the type of decoder, the number of output classes *M*, the (*DR*), the accuracy, the power consumption and the hardware implementation. When input (channel or feature) selection is applied, we report the number of selected inputs followed by the total number.

**Table 1:** Summary of state-of-the-art brain signal decoding hardware implementations.

**Article**			**Inputs**				**Features**			**Task**				**Performance/hardware**		
**#**	**References**	**Year**	**Signal**	**Ch**.	**SR (Hz)**	**Bits**	**Type**	**Method**	**Nb**.	**Type**	**Decoder**	**Classes**	**DR (Hz)**	**Accuracy**	**Power (mW)**	**Implementation**
1	Zhong et al., 2024	2024	EEG	8/16	128	8	–	–	–	Mix^a^	CNN	2	0.5	Task related^a^	0.0905	ASIC (65 nm)
2	Wu et al., 2024	2024	EEG	8	250	10	–	–	–	Drone	TRCA	12	2.5	85.17%	2.46	ASIC (130 nm)
3	Ma et al., 2019	2019	EEG	10/59	100	16	EB	Filter	3	Motor	CNN	3	1	80.5%	25	FPGA
4	Chamanzar et al., 2017	2017	EEG	1/14	128	21	TFC	CWT	1	Motor	Bayesian	2	1	80.5%/68.0%^b^	47.3	FPGA^b^
5	Wang et al., 2016	2016	EEG	4	250	10	EB	FFT	1/16	Eye state	Bayesian	2	0.5	100%	34	DSP
6	Sridhara et al., 2011	2011	EEG	4	256	12	EB	FFT	1	Seizure	Threshold	2	2	–	0.00099	ASIC (130 nm)
7	Shin et al., 2022	2022	Mix^c^	64/256	2,000	10	Mix^d^	Custom	64	Mix^e^	DT	2/6	0.5–4	Task related^e^	0.453	ASIC (65 nm)
8	Malekzadeh-Arasteh et al., 2020	2020	ECoG	32	260	4	EB	Analog	32	–	–	–	–	–	0.0346	ASIC (180 nm)
9	Wang et al., 2019	2019	ECoG	7/32	500	16	EB	Filter	1/14	Motor	Bayesian	2	1.33	87%	150	MCU
10	Agrawal et al., 2016	2016	ECoG	62	1,000	12	PC	PCA	3	Motor	MLP	6	2	82.4%	152	FPGA
11	Won et al., 2014	2014	ECoG	7/32	1,200	8	EB	DCT	42	Motor	Linear	2	3.33	82.9%	0.72	FPGA
12	Chen et al., 2010	2010	ECoG	16	256	9	Mix^f^	Custom	–	Seizure	KNN	2	10	98.2%/97.8%^g^	0.23	ASIC (90 nm)
13	Shaeri et al., 2024	2024	MEA	192/512	20,000	10	DNC	Filter	128	Text	LDA	31	1	91.3%	0.88	ASIC (65 nm)
14	An et al., 2022	2022	MEA	93	10,000	16	SBP	Custom	93	Motor	SSKF	2/4	20	100%	0.581	ASIC (180 nm)
15	Yoon et al., 2021^h^	2021	MEA	1,024	20,000	10	–	–	–	Motor	Threshold	7^h^	4.33^h^	85%^h^	24.7	ASIC (65 nm)
16	Musk and Neuralink, 2019^i^	2019	MEA	3,072	19,300	10	–	–	–	–	-	–	–	–	750	ASIC
17	Boi et al., 2016	2016	MEA	15	25,000	16	–	–	–	Motor	SNN	4	4	70%	4^j^	ASIC (180 nm)
18	Chen et al., 2015	2015	MEA	40/128	10,000	6	–	–	–	Motor	ANN	12	50	99.3%	N/A^k^	ASIC (350 nm)
19	Rapoport et al., 2012	2012	MEA	32	31,250	8	–	–	–	Motor	Threshold	32	11.1	94%	0.537	FPGA

^a^The circuit is tested for different applications including SSVEP, affect monitoring, and mental and motor imagery.

^b^Sensitivity (true positive) vs. Selectivity (true negative). The design is extrapolated to 180 nm ASIC for power estimation.

^c^Tested on EEG and iEEG epilepsy datasets for seizure detection, a μECoG array *in vivo*, and an ECoG finger movement dataset.

^d^The circuit can extract energy band features, phase values and Hjorth parameters.

^e^Epilepsy seizure (95.6% sensitivity-96.8% selectivity), Parkinson tremor (82.6% sensitivity-78.4% selectivity) and finger movement (73.3% accuracy).

^f^Temporal-domain characteristics, Spatial cross-channel correlations, Frequency-domain spectrum features, Nonlinear chaotic values.

^g^Sensitivity (true positive) vs. Specificity (true negative).

^h^No number of decoded classes is reported, neither a decision rate. We assume 7 classes (five for a 2D movement and two for button clicks), which gives a decision rate of 4.3 Hz to explain the reported information transfer rate [480 bits/min Neuralink (2024)] if we assume an accuracy of 85%.

^i^Two configurations with 1,536 (resp. 3,072) electrodes sampled at 19,300 (resp. 18,600) Hz with a 550 (resp. 750) mW power consumption.

^j^The reported power only takes into account the decoding step.

^k^The authors report a power consumption of 0.4μW for the implemented layer, they do not include the acquisition nor the output combination cost.

For the included circuits, the reported power consumption is that of the whole system, which may perform feature extraction, decoding or both. For the Neuralink circuit (Musk and Neuralink, 2019), we report the acquisition power consumption as it dominates the overall power consumption. Note that, although most of the papers describe systems that are implemented on ASICs (ASICs) or FPGAs (FPGAs), some are based on MCUs (MCUs) and DSPs (DSPs). For instance the approach described in Wang et al. (2019) uses a general purpose MCU to run the decoding algorithm. Furthermore, the system in Wang et al. (2016) is based on a DSP. For ASICs, the choice of process technology node (in nm) will have a major influence on the overall power consumption and fabrication cost, which is why it was included in the table. Although the implementation heterogeneity makes it difficult to establish a completely fair comparison, our objective is to discern broad power and performance trade-offs. To our knowledge, this is the first literature analysis that introduces a set of quantitative metrics to compare a broad range of hardware decoders for motor BCIs.

The system in Shin et al. (2022) has two use cases, one where EEG signals are decoded for seizure and Parkinson disease tremor detection and one where ECoG signals are decoded for finger movement classification. Similarly, two configurations are described in Musk and Neuralink (2019), with different numbers of channels (1,536 and 3,072, respectively). The circuit in An et al. (2022) will also appear twice in the next section's graphs as in was tested for two decoding tasks (1-D and 2-D movement). Although the decoding system in Zhong et al. (2024) is tested for different applications, we only focus on its use in motor imagery.

MEA systems sample at a higher rate in order to detect spikes and they generally do not extract intermediate features, but instead, typically decode temporal characteristics (e.g. firing rate) of the spike train (Shaeri et al., 2024; Tanzarella et al., 2023). However, the MEA decoder in An et al. (2022) extracts SBP (SBP) features, the average of a signal filtered in the 300–1,000 Hz band. For ECoG and EEG, the most common extracted features are EBs (EBs) that measure the signal energy at specific frequency ranges. Common frequency bands for these signals include δ(0–4 Hz), θ(4–8 Hz), α(8–13 Hz), β(13–30 Hz), and γ(30–100 Hz) bands (Tam et al., 2019). ECoG signals can also have a high-γ (75–200 Hz) modulation during movement and speech. One approach to extract time-frequency components is to use a wavelet transform (Grossmann and Morlet, 1984). Another system (Agrawal et al., 2016) directly performs a PCA (PCA) on the raw signals, and uses the obtained components as features for decoding. The number and type of features plays a key role in determining the decoding performance and energy consumption of the overall system.

Many of the existing hardware decoders (mainly for EEG and ECoG signals) presented in Table 1 are limited to two output classes and the decision rate (the number of classifications per second) is often below 5 Hz. Emerging motor control applications require more classes to control a higher number of DoFs, for multi-dimensional movements.

Most of the systems use linear decoders, with many using Bayesian models. The system in Wu et al. (2024) performs a TRCA (TRCA), which is an end-to-end decoding method where the signals are matched with templates tailored to the output classes. The circuit in An et al. (2022) uses a SSKF (SSKF) to decode a 1-D or 2-D motor task. Decision trees are another approach for classification, which was used by Shin et al. (2022). Some of the systems use neural network approaches: Zhong et al. (2024), Ma et al. (2019), Boi et al. (2016) and Chen et al. (2015) directly decode the raw input signals, whereas, Agrawal et al. (2016) uses a MLP (MLP) to decode PCA-based extracted from ECoG signals.

It is important to note that all the systems, in Table 1, that perform decoding, use a model that has been trained offline. Two circuits (Wu et al., 2024; Boi et al., 2016) are however able to update the model coefficients based on error feedback.

From Table 1, it can be seen that the algorithmic approaches for brain signal decoding are highly heterogeneous, suggesting there is not yet a consensus on the best approach. All systems report accuracies that are typically over 80%, although the decoding difficulty varies with the different tasks. The reader will note that there is a huge variance in the reported power consumption, and exploring the trends that impact power consumption is the focus of the following sections.

## 3 Comparison metrics for BCI system design and evaluation

In the literature on algorithms for brain signal decoding, the primary metric of interest is usually the classification or decoding accuracy. For real-time motor applications, it is also important that the systems achieve a sufficiently high decision rate to meet the latency requirements of the target application. In order to compare different decoding systems, we consider the simplified reference “decoder” model (Figure 1). We define the IDR (IDR) as the total input bits per second in Equation 1.


(1) $$\begin{array}{l} IDR\left[bits/s\right]=N\times n\left[bits\right]\times SR\left[Hz\right] \end{array}$$

Note, for circuits that perform channel selection, *N* is the number of selected channels.

### 3.1 Analysis based on output classes per second

Controlling a system with a large number of DoFs requires a decoder with more output classes, and of course, achieving high accuracy with a large number of output classes is more difficult. To capture both the task difficulty and the latency requirement, we propose a metric called CpS (CpS), defined in Equation 2.


(2) $$\begin{array}{l} CpS\left[classes/s\right]=M\left[classes\right]\times DR\left[Hz\right] \end{array}$$

**Figure 2:**
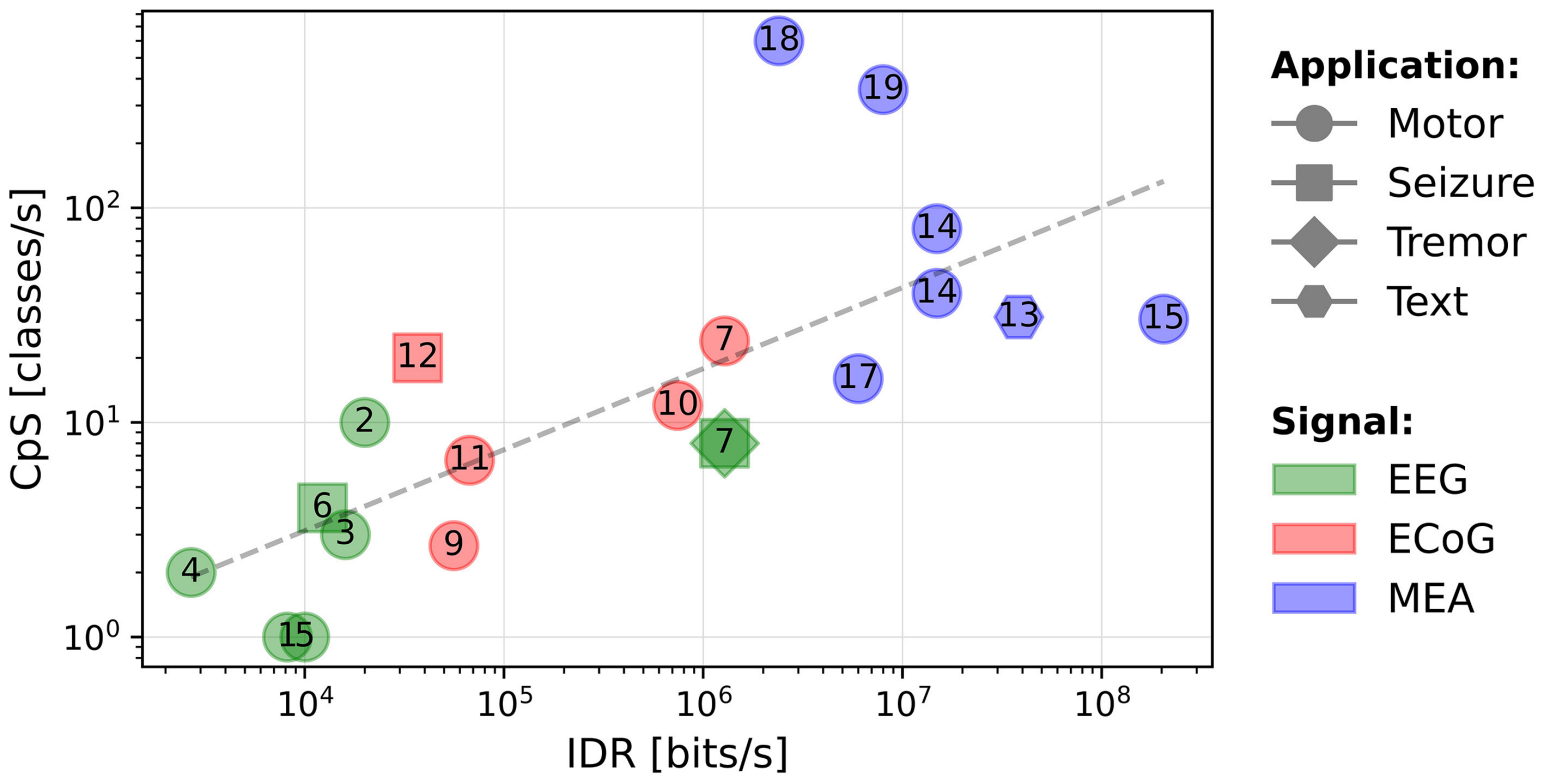
Output classes per second as a function of the input data rate (bits/s). The numbers represent the references numbers in Table 1.

In Figure 2, we present a scatter plot of the systems (log-log scale), where the horizontal axis corresponds to the IDR and the vertical axis corresponds to the output CpS. The shapes correspond to the tested application, the numbers in the shapes refer to the paper references in Table 1, and the colors correspond to the type of signal being decoded.

Despite the systems being highly heterogeneous, we note an increasing tendency for the decoded CpS as function of the IDR. A least-squares fit trendline has been plotted. The regression yields a strong positive correlation (*R*-value = 0.849 for a Spearman's test with a *p*-value of 0.000002). The equation for the trendline is given in Equation 3. This trend may be useful to designers of BCI motor systems to estimate the required IDR for decoding a given number of CpS.


(3) $$\begin{array}{l} CpS=0.097\times {IDR}^{0.38} \end{array}$$

The circuits presented in Chen et al. (2015) and Rapoport et al. (2012) are outliers, achieving a high CpS value as they are able to decode a high number of classes (12 and 32) at a high (11.1 and 50 Hz) using highly parallel compute operations.

### 3.2 Information transfer rate to measure a decoding performance

The ITR (ITR), described in Pierce (1980), is a metric frequently used in BCI systems to describe the amount of information being extracted, taking into account the number of classes *M*, the interval between decisions 1/(the maximum time to produce one new classification for real-time applications), and the achieved accuracy *P*. The formula for computing ITR is shown in Equation 4.


(4) $$\begin{array}{l} ITR\left[bits/min\right]\\ =60\times DR\times \left(\log_{2}M+P\log_{2}P+\left(1-P\right)\log_{2}\left(\frac{1-P}{M-1}\right)\right) \end{array}$$

**Figure 3:**
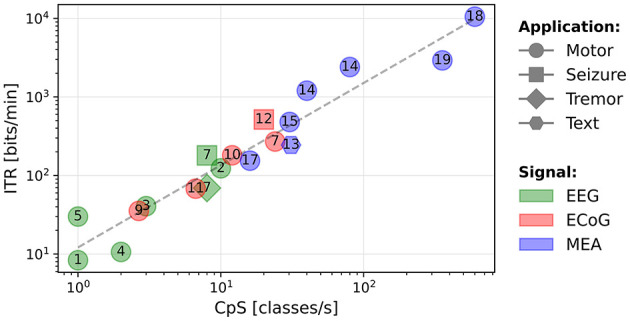
Scatter plot of the ITR (bits/min) as a function of the CpS (classes/s).

Our hypothesis is that systems are not significantly differentiated by accuracy, but that accuracy is mainly a requirement for the system usability. This suggests that, once the accuracy requirement is met for a given task, a high ITR reflects a high and number of classes *M*, thus a high CpS. In Figure 3, we present a scatter plot of ITR vs. CpS. It clearly shows that the two metrics are highly correlated (*R*-value = 0.97 for a Spearman's test with a *p*-value of 10^−11^), showing that accuracy has a marginal influence on the ITR of the systems included in this study. In the remainder of this paper, we will consider the ITR as a metric that reflects the CpS value to compare performance, while validating a sufficient level of accuracy that makes a system usable.

### 3.3 Analysis of circuit power consumption

The CpS and the ITR metrics depend on the rate at which information is output, but they do not provide insight into the computational complexity required to perform the decoding. Given the diversity of decoding approaches, both in terms of the algorithms and the numeric formats, it is difficult to analytically determine the computational cost of an algorithm, for example by counting the number of arithmetic operations. The power consumption of a decoding circuit thus provides an indirect measurement of the computational cost of the decoding technique.

#### 3.3.1 Power vs. IDR

**Figure 4:**
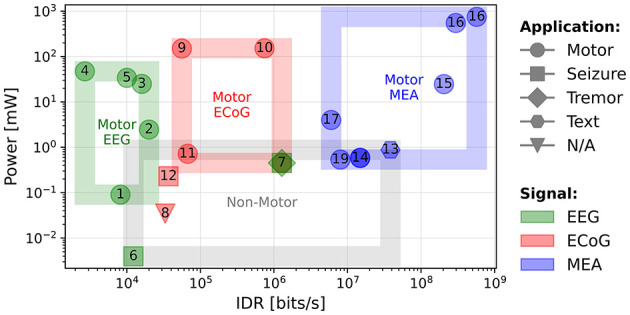
Power consumption (mW) as a function of the IDR (bits/s). Non-motor decoding circuits are grouped together (gray), and motor decoding circuits are grouped according to the signal type: green for EEG, red for ECoG, and blue for MEA.

A scatter plot of the power consumption (in mW) of the circuits vs. the IDR is shown in Figure 4. The plot shows that non-motor applications (epileptic seizure and PD (PD) tremor) using EEG and ECoG signals (gray box) consume less power than those decoding motor intentions. This may be due to the smaller number of features, which require fewer compute operations. For example, only the β-band is extracted in Sridhara et al. (2011) and only the high-γ band in Malekzadeh-Arasteh et al. (2020).

For motor decoding, the circuits can be separated into three groups according to the type of signal. For EEG (green box) and ECoG (red box), the power consumption does not show a strong correlation with the IDR. It implies that the power consumption is determined by the type of processing, including feature extraction, rather than the amount of input data. It is interesting to note that the reported power consumption of the EEG and ECoG decoding circuits is quite similar, while the ECoG circuits decode a much higher IDR. This might be explained by the fact that EEG signals are averaged over a larger number of neurons than ECoG signals (Shokoueinejad et al., 2019), thus requiring more processing to extract relevant information.

In MEA systems, the IDR is significantly higher due to a higher (*SR*). The plot also shows that the power consumption scales with the IDR for these systems. As MEA systems are often based on spike detection and sorting (Zhang and Constandinou, 2023; Yoon et al., 2021; Toosi et al., 2021; Rapoport et al., 2012), the main variable is the number of inputs or the sampling rate, which determines the IDR. This power dependency could also be due to the MEA signals requiring less computation than ECoG and EEG, which means that the majority of the power is used for data acquisition or transmission, parameters that directly scale with the IDR. As the field evolves, and new circuits appear, it will be seen if this trend continues.

#### 3.3.2 Power per channel vs. ITR

The ITR characterizes the amount of useful information output by the system, considering the *DR*, number of output classes *M*, and the decoding accuracy *P*. As discussed in Section 3.2, the ITR primarily reflects the classification rate, as the accuracy of the studied circuits does not vary significantly. The computational cost of an algorithm can be indirectly measured by the overall power consumption. However, the power also depends on the number of channels, especially for systems using MEAs. To better understand the scaling of the power consumption, we consider a normalized metric, the PpC (PpC), to take into account this effect. For embedded applications, the aim is to minimize the circuit power consumption, hence the PpC, given a fixed number of electrodes required to extract the necessary spatial information.

**Figure 5:**
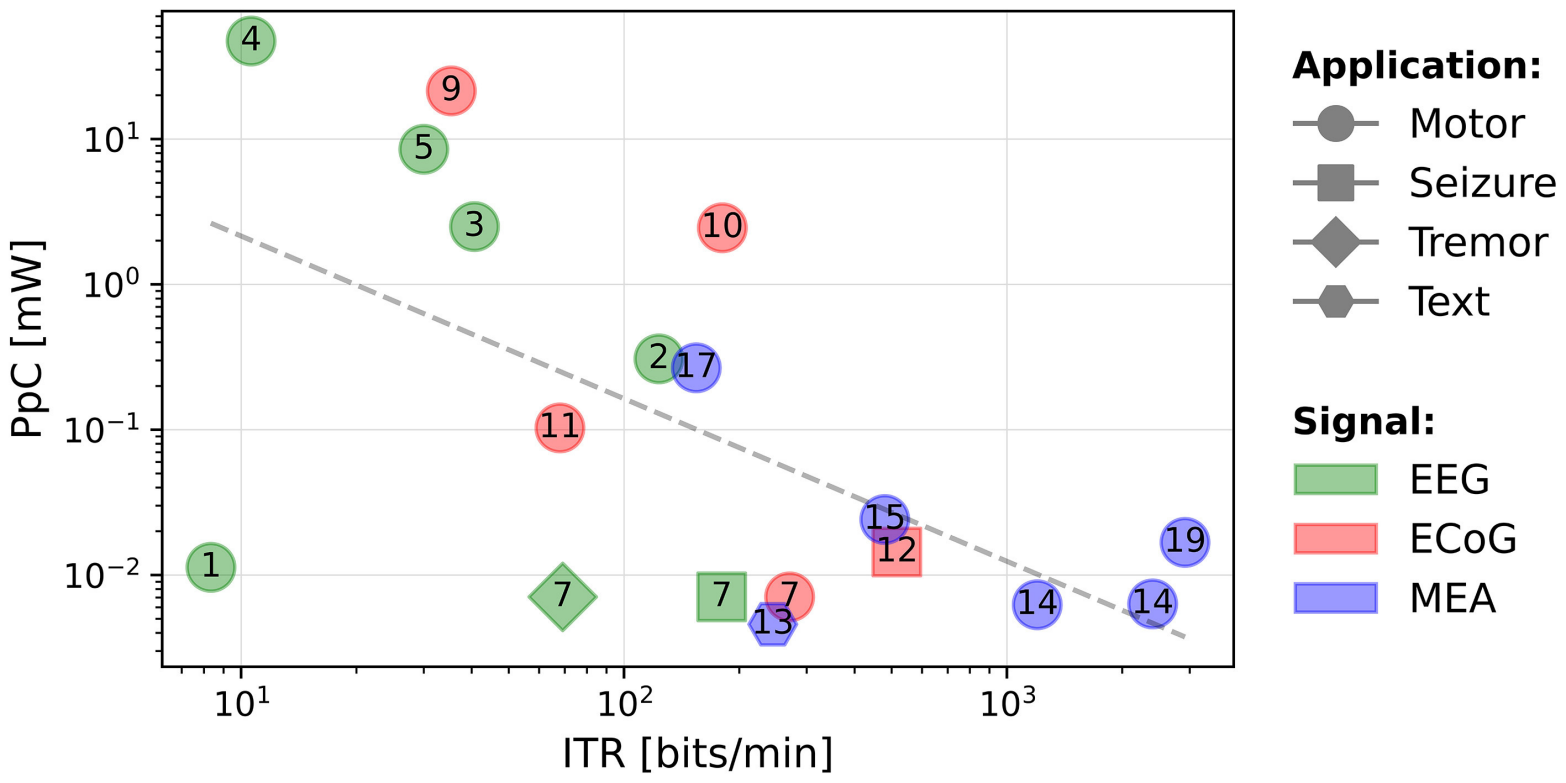
PpC (mW) as a function of the ITR (bits/min).

Figure 5 shows a scatter plot of the PpC vs. the ITR. The best performing circuits are toward the bottom right side of the plot, meaning they provide the most information while using the least power per electrode. In this plot, we note that the circuits in the bottom right region use MEAs. There is no clear difference in the performance between ECoG and EEG systems and the PpC is comparable for both types of signals. In fact, for such systems, the performance depends more on the decoding approach than the signal type. For instance, the circuits that exclusively extract EB (EB) features (Wang et al., 2019; Ma et al., 2019; Chamanzar et al., 2017; Wang et al., 2016) have a lower performance than those extracting a wider range of features (Shin et al., 2022; Chen et al., 2010), or directly decoding raw signals (Zhong et al., 2024; Wu et al., 2024).

We plot the trendline obtained using a least-squares fit on these circuit metrics. A Spearman's test shows a negative correlation (*R*-value = –0.602 with a *p*-value of 0.008) between the ITR and the PpC metric. A way to look at this, is by considering a system that doubles the number of electrodes to increase its ITR. If the power consumption also doubles, the system will only move horizontally on this plot. Whereas, we see that in actual systems, when the ITR doubles, the PpC tends to decrease. One explanation for this trend is that there is always some fixed system energy cost that does not scale with the number of electrodes and hence, as the number of channels increases, this fixed cost is amortized, resulting in a lower PpC. This suggests that systems with a large number of channels can simultaneously benefit from hardware sharing and a large amount of input data, which can enable decoding more classes or potentially improving accuracy. Furthermore, for a fixed number of input electrodes, the best performance is achieved by circuits that extract the most useful information (ITR) with minimal computational cost (PpC). The system (Zhong et al., 2024) is an outlier as the authors' main objective is to reduce the power consumption without necessarily improving the ITR. This plot provides a high-level method to compare BCI decoding circuits, where the best performance is in the lower, right region.

## 4 Power optimizations for BCI circuits

In this section, we present key power optimizations that have been used in the state-of-the-art brain decoding circuits. These can be grouped into three main categories: (i) input selection and reduction, (ii) compute, and (iii) circuit-level optimizations. We focus on optimizations that reduce the number of operations or their power consumption.

### 4.1 Input selection and reduction

Many hardware decoders rely on offline calculations and optimizations to simplify the compute required for online decoding. This offline processing is often used to reduce the amount of data that must be processed for inference. The simplifications are applied to either the raw signals or the extracted features.

#### 4.1.1 Electrode selection

**Figure 6:**
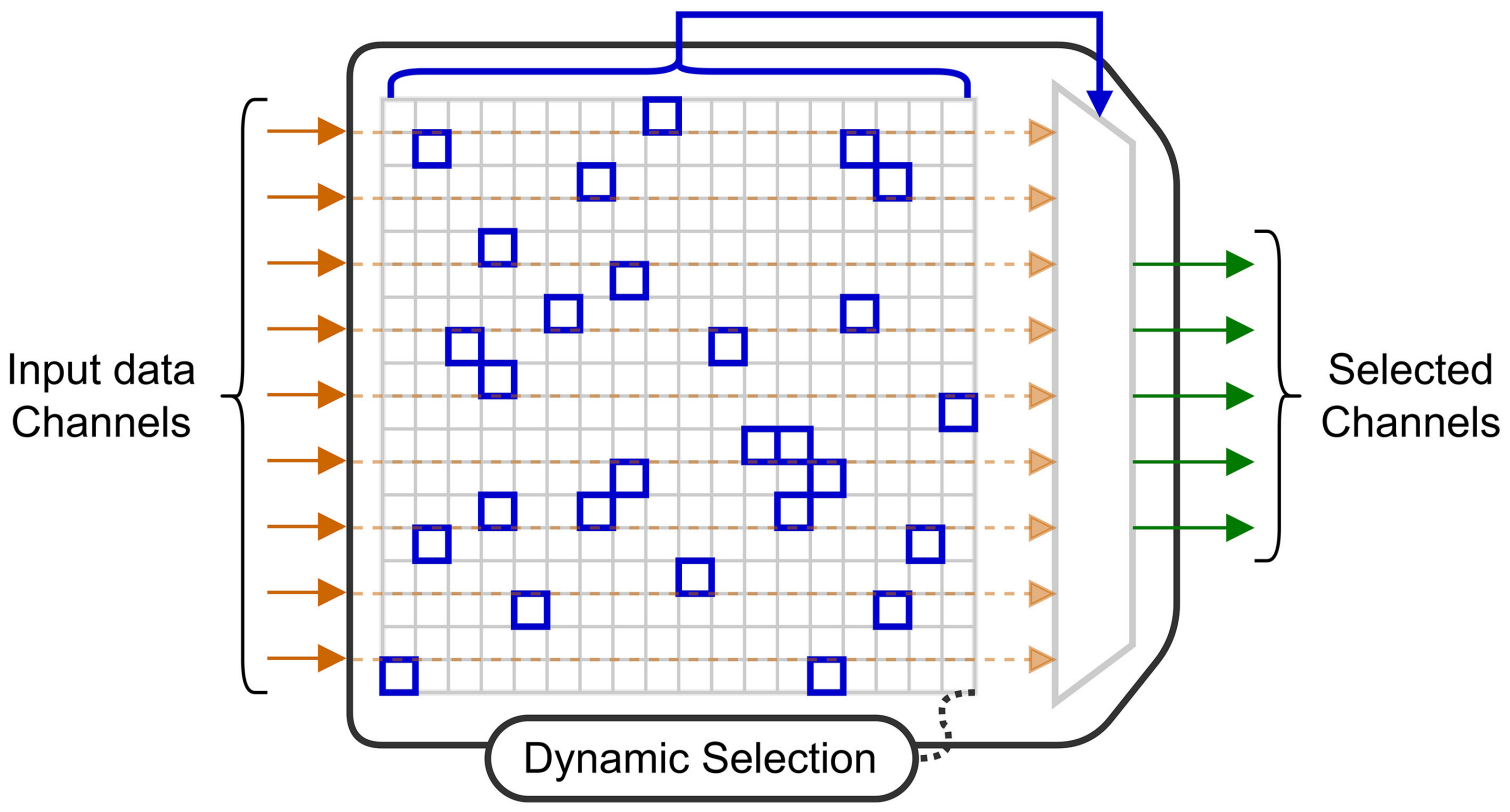
Block diagram of a switch matrix for channel selection.

A highly effective technique to reduce power consumption is to only collect data from electrodes that provide useful information for the current decoding task. The circuit in Shin et al. (2022), uses a 256-electrode grid to decode stimulation patterns. In the training mode, four modules are used to convert the signals to a digital format so they can all be used by an offline training algorithm. This algorithm determines the probabilistic weights of a DT (DT) decoder, and a subset with the 64 best electrodes (selected using a 16 × 16 switch matrix) to be used when performing each step of the online classification. With this approach, the circuit benefits from the potential information from a large number of electrodes, while limiting the power required for signal acquisition and decoding, as three of the four modules are powered-off. Similarly, the MiBMI circuit (Shaeri et al., 2024) and the Neuralink chip (Yoon et al., 2021) both include a switch matrix to dynamically select input channels for decoding. The diagram in Figure 6 shows an acquisition system for which a selection algorithm dynamically selects a subset of electrodes for inference.

Electrode selection has also been studied for online training of BCI algorithms. A penalized method is introduced in Moly et al. (2023) to obtain sparse decoding models when using a Recursive Exponentially Weighted N-way Partial Least Squares algorithm for training. The algorithm is based on a PARAFAC tensor decomposition (Harshman and Lundy, 1994) where the objective function to minimize is the quadratic error between the labels and predictions. With this penalized method, an additional cost term, proportional to the number of electrodes, is added to the objective function. By applying this penalization, a model with 75% sparsity was obtained, meaning that only one out of every four electrodes was required. The sparse model achieved a cosine similarity close to that of the original model, thus reducing compute time and memory consumption with no significant loss in accuracy.

Other techniques can be used for electrode selection. The authors in Wang et al. (2019) select a subset of the available electrodes that maximizes the contrast between the classified states. By applying this technique, they were able to empirically select the seven best channels out of the 32 available to be used for decoding. Other criteria can be used for channel selection. For instance, the Fisher criterion was used in Won et al. (2014) to determine the top seven channels to be used for decoding. The authors in Zhong et al. (2024) and Chamanzar et al. (2017) select the best channels as the ones achieving the highest decoding accuracy for a specific task. Finally, the selection can also be based on previous works that have determined spatial localization of task-related brain signals, such as was done in Ma et al. (2019). More precisely, in Zhong et al. (2024), only eight electrodes are selected to be placed at specific positions on a head-strap to support a variety of mental tasks. Then, a subset of these electrodes is used for each task, which minimizes the amount of input data to process, hence the power consumption.

Although less common, it is possible to combine inputs from different electrodes such as the approach in Boi et al. (2016) where data from 15 different channels is merged to create a combined spike train. This optimization makes it possible to use a reduced number of synapses per neuron in the downstream SNN (SNN) decoder.

#### 4.1.2 Feature reduction and selection

**Figure 7:**
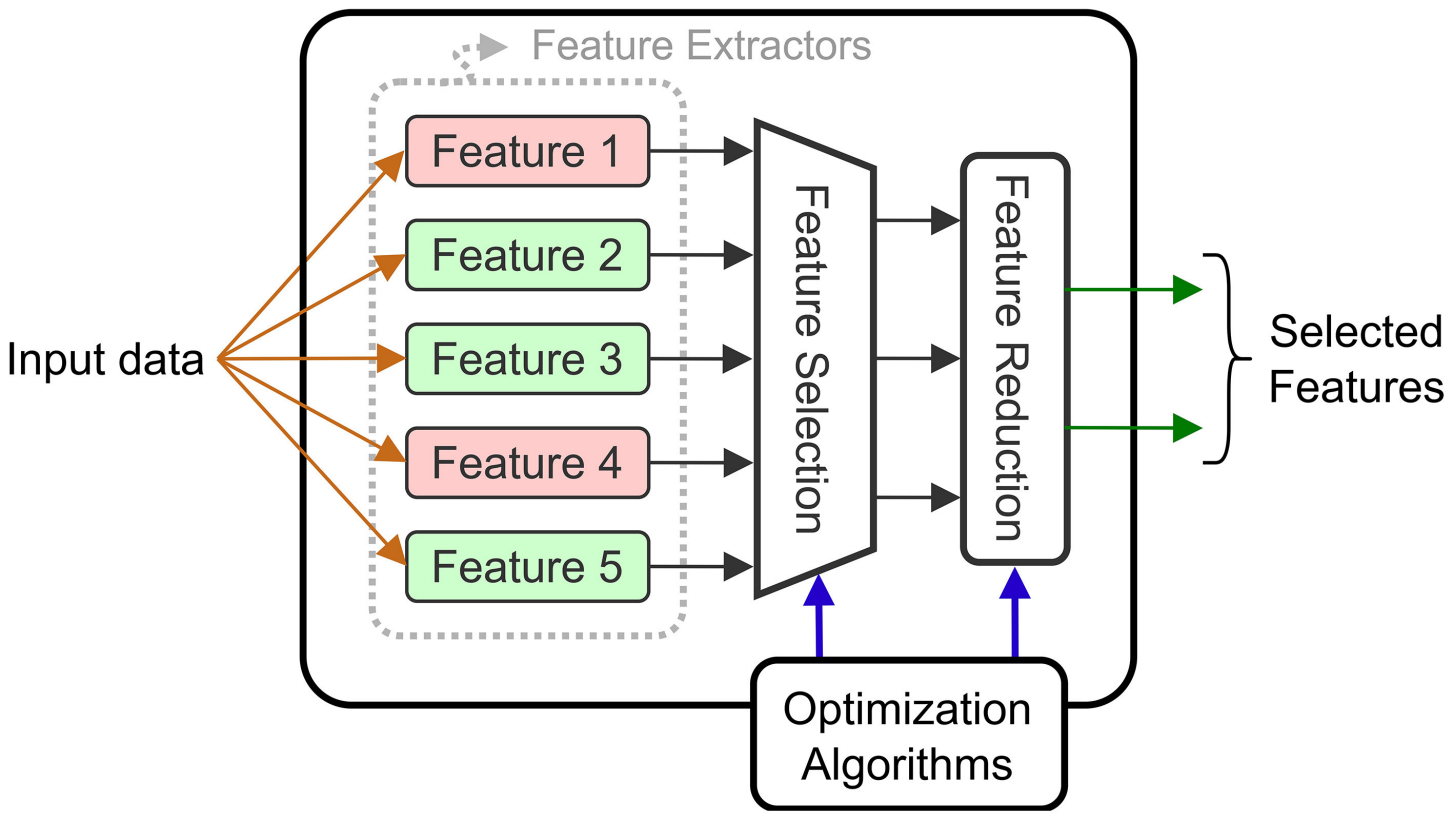
Block diagram of a feature extraction block supporting *selection* and *reduction*.

Input selection is not limited to the raw input signals, but can also be applied for the extracted features. When we refer to *feature selection*, it means that certain features that were initially identified, are completely eliminated. When *feature selection* is applied, there is a double benefit: the eliminated features no longer need to be computed, which reduces energy, and the size of the downstream decoder is reduced. *Feature reduction* consists of combining the computed features into a reduced intermediate representation that is used by the decoder. However, the energy savings are limited to the decoder level as all the features are computed and then combined. Both techniques are common in the BCI literature, and they can be combined with electrode selection techniques. Figure 7 shows a diagram of a general circuit that uses a *feature selection* module to extract features adapted to a given task. The dynamically chosen features are then combined (projected in a latent space) using a *feature reduction* block.

The authors in Wang et al. (2019) perform *feature reduction* using a class-wise PCA (retaining 92% of variance per class) to extract linear combinations of two selected EBs (one is in the α and β bands, the other in the high-γ band). These linear combinations are then reduced to a single value using LDA (LDA), and this value is fed to a Bayesian decoder for ECoG motor (grasp) decoding. In earlier work, the same authors (Wang et al., 2016) also implemented a similar approach on a DSP, however, the PCA was done across all the data, using a higher threshold (99.7%) for the retained variance.

The epileptic seizure detection SoC (SoC) in Chen et al. (2010) has a hardware module to extract multiple types of features, followed by a dimension reduction unit that combines the extracted features for inference. The reduction unit is programmable and could implement a PCA that has been computed offline. The *feature reduction* step contributes to the low-power implementation, and enables the classification using a micro-processor.

In Chamanzar et al. (2017), a different approach is used based on an adaptive WT (WT). The key idea is to generate, during the training phase, a special template that identifies the onset of movement intent. For each of the two classes, the FFT (FFT) of the input signal is calculated. Using the Fisher Discriminant Ratio, they identify a small set of frequency bands that best discriminate between the classes. The iFFT (iFFT) of these selected frequency bands is computed, thus providing a template which is used for detection. Using this approach for designing a custom filter, requires less power than extracting each of the required frequency components, which is an interesting approach for *feature selection*. The Fisher criterion is also used in Won et al. (2014) to select the six best frequency bands for a decoder using a DCT (DCT) to extract EB features.

Another adaptive method is applied in Malekzadeh-Arasteh et al. (2020) where the circuit extracts power envelopes in the gamma band using band-pass filters. Since the specific frequency range varies between individuals, a dual-mode architecture is proposed with two operating regimes: FB (FB) and BB (BB) modes. In the FB mode, the circuit captures the raw brain signals in the analog front-end layer with high resolution (8–10 bits sampled at 13 KHz), and the digital back-end uses these signals to compute patient specific weights, for filters in the neural pre-processing unit. For inference, the system operates in the BB mode with lower ADC resolution (3–4 bits sampled at 260 Hz). In this mode, power-band features are extracted using the previously calibrated filters.

In addition to reducing the number of features, off-chip processing can help in selecting the set of extracted features when the circuit offers a configurable feature extraction engine. As the circuit in Shin et al. (2022) is meant to be used for different applications, a set of varied features is extracted from the selected electrodes. These consist of: LL (LL), LMP (LMP), HFO (HFO), Hjorth parameters [ACT (ACT), MOB (MOB) and COM (COM)], PAC (PAC), PLV (PLV) and EB (EB) extracted using band-pass filters. The authors reduce power using an energy-aware objective function for training that minimizes cross-entropy while also penalizing the use of features requiring a high power consumption. This method reduces power by 64% while resulting in less than a 2% loss in decoding accuracy.

The circuit in Shaeri et al. (2024) extracts DNC (DNC) features to decode handwritten characters. These features correspond to spike rates at different time windows measured from different electrodes. An offline algorithm selects the 64 DNCs that best distinguish a given class, and these are fed into a LDA decoder for classification. *Feature selection* reduces the number of operations by a factor of 320 × and the memory size by 7.8 × .

All the *feature selection* and *reduction* methods aim to take into account an optimized set of features (often determined offline) that reduces the required compute power for inference. The optimizations can be combined with other methods to further reduce the power consumption of BCI systems.

### 4.2 Compute optimization

As seen above, reducing the number of electrodes and the number of features directly reduces the number of compute operations, thus overall power. However, the power also depends on the way these operations are implemented in hardware. We review some techniques that have been used to efficiently implement specific compute operations.

#### 4.2.1 Approximate compute operations

Another approach to reduce power consumption is to reduce the precision of the decoding computations, often using approximate computing techniques. For example, to implement the feature extraction methods efficiently in hardware, the authors in Shin et al. (2022) approximate the theoretical definitions for the features, with simplified equations. For example, the *L*_2_-norm is replaced by *L*_1_ when computing the HPs (HPs), and the phase is computed using a linear arctangent approximation with a LUT (LUT) error correction. These simplifications yield features similar to the theoretical ones computed in Matlab (with a median correlation above 0.9) but at a lower compute cost. Similarly, the authors in Rapoport et al. (2012) use a low-power spike pattern matching based on a logical *AND* comparison between the spike counts instead of a costly multiplication.

The circuit in Agrawal et al. (2016) implements a simplified PCA algorithm, described in Chen et al. (2008), which uses only adders and multipliers. The norm and division operators are simplified to additions and multiplications using fixed-point arithmetic. In addition, a log-sigmoid activation function is implemented using a LUT, taking input values between –5 and 5 expressed as 9-bit values.

Moving from floating-point to fixed-point arithmetic is common in low-power decoding circuits (Shin et al., 2022; An et al., 2022; Ma et al., 2019; Sridhara et al., 2011). The circuit in Won et al. (2014) achieves a 82.9% (resp. 75.7%) accuracy when using fixed-point (resp. floating point) operations. The authors suggest that, in some cases, the quantization for fixed-point arithmetic can remove random ECoG noise, thus yielding a better decoding accuracy. In addition to using fixed-point arithmetic, the chip in Yoon et al. (2021) supports custom precision to save power. They report a “99.7% match” compared to the original approach with floating-point arithmetic.

More system-level approximations can be applied to further reduce the power consumption. For example, the circuit in Zhong et al. (2024) implements a teacher-student CNN (CNN) approach for decoding. It consists of two CNNs with different architectures: the teacher, a large model with high power consumption and high decoding accuracy, and the student, a smaller model that consumes 70% less energy, but achieves a 14% lower accuracy. During the decoding process, only the student model is turned on when the same state is decoded. The system switches between both models only when a state transition is detected with a low confidence level. This level is defined according to a confusion matrix between the decoders' outputs. The hybrid architecture reduces the overall energy consumption by 55%. The system also takes advantage of the EEG signal sparsity for further power reduction.

#### 4.2.2 Merging operations

Another technique to reduce the number of compute operations is to merge multiple operations in the decoding equations. In other words, certain steps can be reordered or combined before being implemented in hardware.

An interesting combination method is described in Wu et al. (2024) where SSVEP (SSVEP) are decoded using a TRCA (TRCA) algorithm. The processing combines three main steps: pre-processing using a temporal filter, feature extraction with spatial filtering, and pattern recognition with a SSVEP template signal. The steps are mapped into a single matrix for fast computation. In this paper, the decoder's updates are directly applied to the combined matrix, based on a feedback signal.

In Won et al. (2014) a DCT is applied to extract the signal energy at given frequencies. The DCT of a signal {*x*_*n*_, *n*∈⟦0, *N*−1⟧} is defined by Equation 5.


(5) $$\begin{array}{l} X_{m}=\overset{N-1}{\underset{n=0}{\sum }}x_{n}\cos\left[\frac{m\pi }{N}\left(n+\frac{1}{2}\right)\right] \end{array}$$

Where *m* is a scaling parameter fixing a frequency and *N* the length of the input signal. To reduce the number of multiplications, the authors propose a reduced-resolution quantization of the cosine function in Equation 5. In fact, by using 11 levels of quantization ( ≤ 4 bits), the system only requires 11 multiplications to compute the DCT, regardless of the size of the input signal. More precisely, the input signal samples are divided (using a LUT based on their indexes) into 11 sets and the elements of each set are summed before multiplying with the corresponding quantized cosine coefficient. This technique can be more generally applied to any large sum of products, assuming one of the factors can be coarsely quantized.

### 4.3 Circuit-level optimizations

In addition to input and compute optimizations, some circuits propose circuit-level improvements. These can include analog implementations of the compute operations or other custom low-power circuit techniques.

#### 4.3.1 Analog approaches

In BCI systems, input signals are analog. While digital circuits offer greater flexibility and ease of design than their analog counterparts, they require multi-channel, low noise ADCs at a sampling rate ranging from a few hundreds to a few thousands of Hz to provide the digitized input data. This section examines power-saving optimizations enabled by analog integrated circuits, focusing on two types: analog feature extraction and analog-based accelerators for brain signal decoding.

A typical processing task is energy extraction over one or several frequency bands. Performing this function in the analog domain reduces the amount of information that is sent to the decoding stage, and hence reduces the power consumption. For instance, in Malekzadeh-Arasteh et al. (2020), the authors propose a low-power Base Band mode, which consists of a single tunable analog band-pass Gm-C filter followed by a multiplier and a low-pass filter to capture, for each channel, gamma-band power envelopes which are then digitized. This low-power mode only consumes 1.05 μW/ch including 0.205 μW/ch for feature extraction. Another example is a bidirectional BCI (Liu et al., 2017) that features an analog per-band energy estimator and a programmable Proportional—Integral—Derivative (PID) control block providing feedback through a stimulator. The features are extracted by a programmable band-pass Gm-C filter bank, and then digitized at a low sampling rate. With this analog approach, the feature extraction only consumes 56 μW/ch. In Zhang et al. (2011), the authors use four 4th order switched capacitor filters, multipliers and integrators to extract the signal energy over four frequency bands. This energy extraction consumes 3.1 μW/ch, and 10.8 μW/ch when accounting for bias, clocks and amplifiers. In Lim et al. (2020), SBP (SBP) is extracted from a neural signal sensed by an intracortical microelectrode and rectified in the analog domain. The signal goes through an integrate-and-fire circuit that controls a near infrared LED, thus transmitting the energy information as a blinking rate. This purely analog approach only consumes 0.74 μW/ch for SBP detection and enables downstream accurate finger position and velocity decoding.

Analog approaches can also be used in accelerators for brain signal decoding to achieve higher computing power efficiency. In Chen et al. (2015), the authors propose an analog decoder that consists of a dense neural network with a single hidden layer. Neurons in this layer are implemented using current-controlled oscillators. Operating in the sub-threshold region, the oscillator matrix consumes 0.4 μW.

Looking forward, neuromorphic architectures aim to replicate the behavior of biological synapses and neurons on silicon, achieving power-efficient sparse computations. This approach is particularly well suited for BCI applications, where the goal is to process and adapt to actual brain signals. In Wu et al. (2024), an analog decoding accelerator is implemented using a memristor array to perform the matrix-vector multiplications of the TRCA algorithm. The circuit is used to perform a 12-class decoding task while consuming 2.46 mW. The system can be incrementally updated by integrating feedback responses in a closed-loop system to maintain performance over time. The authors in Boi et al. (2016) propose an end-to-end neuromorphic decoder that takes spikes as input and decodes four classes. It can be trained online using spike-timing-dependent plasticity and the local Hebbian update rule, consuming about 4 mW in their experiments. The last two circuits serve as proof of concept for neuromorphic architectures, paving the way for future adaptive low-power BCIs.

#### 4.3.2 Other power optimizations

Circuit optimizations can also involve custom blocks with specific properties. For example, a custom SRAM (SRAM) in Sridhara et al. (2011) using a sub-threshold 6T design is used with the decoder. It provides an ultra-low power retention mode during which it has a leakage power of only 28 fW/bit. This circuit also focuses on improving the efficiency of the power delivery system (DC/DC converter). The MiBMI circuit's (Shaeri et al., 2024) power consumption is reduced by an additional factor of 12.9 × through memory sharing by using variable size memory blocks across inputs and classes. Clearly, such methods can be combined with the compute and algorithmic techniques from the previous sections.

### 4.4 Optimization summary

We have analyzed all of the studied circuits to identify which of the different power optimization techniques were employed and the result is summarized in Table 2, where for each of the circuits used for brain signal decoding, we list the applied optimizations.

**Table 2:** Summary of BCI circuit state-of-the-art summarizing the applied optimizations.

**Article**			**Input selection/reduction**		**Compute optimization**		**Circuit-level**	
**References**	**Year**	**Signal**	**Channel**	**Feature**	**Approximation**	**Merging**	**Analog**	**Custom**
Zhong et al., 2024	2024	EEG	✓		✓			
Wu et al., 2024	2024	EEG				✓	✓	
Ma et al., 2019	2019	EEG	✓		✓			
Chamanzar et al., 2017	2017	EEG	✓	✓				
Wang et al., 2016	2016	EEG		✓				
Sridhara et al., 2011	2011	EEG			✓			✓
Shin et al., 2022	2022	Mix	✓	✓	✓			
Malekzadeh-Arasteh et al., 2020	2020	ECoG		✓			✓	
Wang et al., 2019	2019	ECoG	✓	✓				
Agrawal et al., 2016	2016	ECoG			✓			
Won et al., 2014	2014	ECoG	✓	✓	✓	✓		
Chen et al., 2010	2010	ECoG		✓				
Shaeri et al., 2024	2024	MEA	✓	✓				✓
An et al., 2022	2022	MEA			✓			
Yoon et al., 2021	2021	MEA	✓		✓			
Boi et al., 2016	2016	MEA	✓				✓	
Chen et al., 2015	2015	MEA					✓	
Rapoport et al., 2012	2012	MEA			✓			

The summary shows that the most common optimization techniques used in BCI circuits are those based on input selection, feature selection and reduction, and compute approximation. The input selection and reduction methods often consist of using an offline algorithm to only select inputs that are relevant. Compute approximations, such as quantization of the numeric values and the use of simplified functions, are also widely used. Fewer works have employed circuit optimization techniques as these require access to a custom silicon design flow.

## 5 Discussion

This study presents insights into the performance of BCI decoding circuits. The introduced metrics give empirical rules for circuit design for brain signal decoders. In addition to this, different optimization techniques can be combined to reduce power consumption while maximizing the extraction of useful information for decoding.

The CpS metric was shown to be correlated to the IDR, and we provided the coefficients of a trendline (Equation 3). For a given application, the required and the number of classes are known, thus the CpS can be easily calculated. The required IDR can be estimated using our trendline, and can be used to ensure the number of electrodes, the and ADC accuracy of the acquisition system are sufficient. Of course, there are limitations for each parameter that must be respected: for example there is no value in increasing the beyond the intrinsic frequency of the input signals.

In addition, we showed that the overall power consumption does not appear to scale with the IDR for EEG and ECoG systems. This suggests there is a potential for system power reduction, through the optimization of the compute operations for the decoding algorithms. For MEA systems, as the main power consumption is associated with the signal acquisition, this is where optimizations are most effective. Furthermore, for all systems, techniques such as quantization, grouping of operations and analog computing have been shown to be useful in reducing power consumption.

The study summarized input selection methods that can save power by either processing less data or by extracting fewer features. The selection process can be done offline using objective functions that penalize costly features (Shin et al., 2022) or the number of electrodes during model training (Moly et al., 2023). Combining a channel-penalizing algorithm with hardware that can power-off the circuitry associated with unused electrodes (Shin et al., 2022; Yoon et al., 2021) is an effective power-saving technique.

State-of-the-art brain signal decoders often focus primarily on improving the decoding performance. The ITR, which depends on the accuracy, the number of classes (task difficulty) and the *DR*, is also a widely adopted performance metric for real-time applications. Our work shows that the ITR is mainly dominated by the and the number of classes, which define the CpS metric, and that the accuracy is rather a requirement than a differentiating metric when comparing BCI decoders. We also demonstrate that it is possible to improve the ITR while reducing the PpC.

This is possible through hardware sharing (e.g. using a switch matrix) when using more channels, and through improving the efficiency of the decoder using techniques such as those discussed in Section 4. Furthermore, using *feature selection* and *feature reduction*, the computation load can be reduced while also improving accuracy, as the representation in the latent space can be easier to classify. By plotting different BCI circuits in this two-dimensional space (Figure 5), it is possible to compare both their power efficiency and decoding capability.

Our analysis of existing circuits suggests that circuits that extract a wider range of features (Shin et al., 2022; Chen et al., 2010) perform better (higher ITR and lower PpC) than circuits exclusively relying on EB features such as Ma et al. (2019), Wang et al. (2016); Sridhara et al. (2011), Wang et al. (2019), and Won et al. (2014). In fact, there is a limit to the amount of information available in EB features. Extracting a wider range of features can require higher power consumption if no optimizations are applied. It is hence important to select the relevant subset of features by either using offline selection methods or by dynamically adapting the extracted feature set to the decoding task. Furthermore, using compute approximations when extracting features (Shin et al., 2022; Agrawal et al., 2016; An et al., 2022; Ma et al., 2019; Sridhara et al., 2011; Won et al., 2014) can also reduce the processing power consumption by reducing the power cost per extracted feature.

## 6 Conclusion

In this paper, we have reviewed a broad range of recent BCI circuits and compared them using quantitative metrics. This analysis can help identify which type of brain signals are appropriate for an application, as well as establishing an estimate of the IDR, for a given CpS. Our graphs show that MEA systems achieve higher CpS than either ECoG or EEG. Our findings also suggest that reducing the power consumption does not necessarily mean decreasing the BCI decoding performance, measured with metrics such as the ITR. In fact, we observed that in existing circuits, there is a negative correlation between the PpC and the ITR. This suggests that there remain significant opportunities to simultaneously optimize both performance metrics.

Based on our review, we have identified and summarized the techniques that BCI decoders employ to reduce power consumption. The key to reduced power consumption is to process the minimum amount of data, which requires input selection and reduction, quantization of data and low-cost arithmetic operations. In addition to reducing power consumption, careful feature selection, both by type of feature and the use of lower dimensional representations, can also reduce power while preserving a high decoding performance. It is interesting to note that no single system employs all the known power reduction techniques, which also suggests there remain opportunities for further improvements.

Although our study is limited to BCI circuits that were designed for motor decoding, or which have the potential to be used for such applications, the methodology and metrics could be extended to brain signal decoders for other applications (e.g. seizure detection, speech). The framework of metrics that we have presented facilitates performance comparisons, and we expect that future systems will exceed the performance of the current ones that were studied, allowing portable BCIs to be used in rehabilitation and assistive applications. It is our hope that this broad review and analysis of the techniques used in BCI decoding circuits will help designers of future systems.

## References

[B1] Agrawal M. Vidyashankar S. Huang K. (2016). “On-chip implementation of ECoG signal data decoding in brain-computer interface,” in 2016 IEEE 21st International Mixed-Signal Testing Workshop (IMSTW) (Sant Feliu de Guixols: IEEE), 1–6. 10.1109/IMS3TW.2016.7524225

[B2] An H. Nason-Tomaszewski S. R. Lim J. Kwon K. Willsey M. S. Patil P. G.. (2022). A power-efficient brain-machine interface system with a sub-MW feature extraction and decoding ASIC demonstrated in nonhuman primates. IEEE Trans. Biomed. Circuits Syst. 16, 395–408. 10.1109/TBCAS.2022.3175926 35594208 PMC9375520

[B3] Behrenbeck J. Tayeb Z. Bhiri C. Richter C. Rhodes O. Kasabov N.. (2019). Classification and regression of spatio-temporal signals using NeuCube and its realization on SpiNNaker neuromorphic hardware. J. Neural Eng. 16:026014. 10.1088/1741-2552/aafabc 30577030

[B4] Benabid A. L. Costecalde T. Eliseyev A. Charvet G. Verney A. Karakas S.. (2019). An exoskeleton controlled by an epidural wireless brain–machine interface in a tetraplegic patient: a proof-of-concept demonstration. Lancet Neurol. 18, 1112–1122. 10.1016/S1474-4422(19)30321-7 31587955

[B5] Bin Altaf M. A. Zhang C. Yoo J. (2015). “21.8 A 16-ch patient-specific seizure onset and termination detection SoC with machine-learning and voltage-mode transcranial stimulation,” in 2015 IEEE International Solid-State Circuits Conference - (*ISSCC) Digest of Technical Papers* (San Francisco, CA: IEEE), 1–3. 10.1109/ISSCC.2015.7063092

[B6] Boi F. Moraitis T. De Feo V. Diotalevi F. Bartolozzi C. Indiveri G.. (2016). A bidirectional brain-machine interface featuring a neuromorphic hardware decoder. Front. Neurosci. 10:563. 10.3389/fnins.2016.00563 28018162 PMC5145890

[B7] Chamanzar A. Shabany M. Malekmohammadi A. Mohammadinejad S. (2017). Efficient hardware implementation of real-time low-power movement intention detector system using FFT and adaptive wavelet transform. IEEE Trans. Biomed. Circuits Syst. 11, 585–596. 10.1109/TBCAS.2017.2669911 28534785

[B8] Chen T.-C. Lee T.-H. Chen Y.-H. Ma T.-C. Chuang T.-D. Chou C.-J.. (2010). “1.4μW/channel 16-channel EEG/ECoG processor for smart brain sensor SoC,” in 2010 Symposium on VLSI Circuits (Honolulu, HI: IEEE), 21–22. 10.1109/VLSIC.2010.5560258

[B9] Chen T.-C. Liu W. Chen L.-G. (2008). “VLSI architecture of leading eigenvector generation for on-chip principal component analysis spike sorting system,” in 2008 30th Annual International Conference of the IEEE Engineering in Medicine and Biology Society (Vancouver, BC: IEEE), 3192–3195. 10.1109/IEMBS.2008.4649882 19163385

[B10] Chen X. Yu Y. Tang J. Zhou L. Liu K. Liu Z.. (2022). Clinical validation of BCI-controlled wheelchairs in subjects with severe spinal cord injury. IEEE Trans. Neural Syst. Rehabil. Eng. 30, 579–589. 10.1109/TNSRE.2022.3156661 35259107

[B11] Chen Y. Yao E. Basu A. (2015). “A 128 channel 290 GMACs/W machine learning based co-processor for intention decoding in brain machine interfaces,” in 2015 IEEE International Symposium on Circuits and Systems (ISCAS) (Lisbon: IEEE), 3004–3007. 10.1109/ISCAS.2015.7169319

[B12] Chua A. Jordan M. I. Muller R. (2022). SOUL: an energy-efficient unsupervised online learning seizure detection classifier. IEEE J. Solid-State Circuits 57, 2532–2544. 10.1109/JSSC.2022.3172231

[B13] Fleming J. E. Benjaber M. Toth R. Zamora M. Landin K. Kavoosi A.. (2023). “An embedded intracranial seizure monitor for objective outcome measurements and rhythm identification,” in 2023 45th Annual International Conference of the IEEE Engineering in Medicine & *Biology Society (EMBC)* (Sydney, NSW: IEEE), 1–6. 10.1109/EMBC40787.2023.10340850 PMC7615373 38083730

[B14] Fraczek T. M. Ferleger B. I. Brown T. E. Thompson M. C. Haddock A. J. Houston B. C.. (2021). Closing the loop with cortical sensing: the development of adaptive deep brain stimulation for essential tremor using the activa PC+S. Front. Neurosci. 15:749705. 10.3389/fnins.2021.749705 34955714 PMC8695120

[B15] Greiner N. Barra B. Schiavone G. Lorach H. James N. Conti S.. (2021). Recruitment of upper-limb motoneurons with epidural electrical stimulation of the cervical spinal cord. Nat. Commun. 12:435. 10.1038/s41467-020-20703-1 33469022 PMC7815834

[B16] Grossmann A. Morlet J. (1984). Decomposition of hardy functions into square integrable wavelets of constant shape. SIAM J. Math. Anal. 15, 723–736. 10.1137/0515056

[B17] Guirgis M. Chinvarun Y. Carlen P. L. Bardakjian B. L. (2013). “The role of delta-modulated high frequency oscillations in seizure state classification,” in 2013 35th Annual International Conference of the IEEE Engineering in Medicine and Biology Society (EMBC) (Osaka: IEEE), 6595–6598. 10.1109/EMBC.2013.6611067 24111254

[B18] Hammer J. Fischer J. Ruescher J. Schulze-Bonhage A. Aertsen A. Ball T.. (2013). The role of ECoG magnitude and phase in decoding position, velocity, and acceleration during continuous motor behavior. Front. Neurosci. 7:200. 10.3389/fnins.2013.00200 24198757 PMC3814578

[B19] Harshman R. A. Lundy M. E. (1994). PARAFAC: parallel factor analysis. Comput. Stat. Data Anal., 18, 39–72. 10.1016/0167-9473(94)90132-5

[B20] Herff C. Diener L. Angrick M. Mugler E. Tate M. C. Goldrick M. A.. (2019). Generating natural, intelligible speech from brain activity in motor, premotor, and inferior frontal cortices. Front. Neurosci. 13:1267. 10.3389/fnins.2019.01267 31824257 PMC6882773

[B21] Jang M. Yu W.-H. Lee C. Hays M. Wang P. Vitale N.. (2023). “A 1024-channel 268 nW/pixel 36x36 μm2/ch data-compressive neural recording IC for high-bandwidth brain-computer interfaces,” in 2023 IEEE Symposium on VLSI Technology and Circuits (VLSI Technology and Circuits) (Kyoto: IEEe), 1–2. 10.23919/VLSITechnologyandCir57934.2023.10185288 PMC11463976 39391047

[B22] Jarosiewicz B. Morrell M. (2021). The RNS system: brain-responsive neurostimulation for the treatment of epilepsy. Expert Rev. Med. Devices 18, 129–138. 10.1080/17434440.2019.1683445 32936673

[B23] Kartsch V. Tagliavini G. Guermandi M. Benatti S. Rossi D. Benini L.. (2019). BioWolf: A Sub-10-mW 8-Channel advanced brain–computer interface platform with a nine-core processor and BLE connectivity. IEEE Trans. Biomed. Circuits Syst. 13, 893–906. 10.1109/TBCAS.2019.2927551 31295119

[B24] Kavoosi A. Toth R. Benjaber M. Zamora M. Valentín A. Sharott A.. (2022). “Computationally efficient neural network classifiers for next generation closed loop neuromodulation therapy - a case study in epilepsy,” in 2022 44th Annual International Conference of the IEEE Engineering in Medicine & *Biology Society (EMBC)*, 288–291. 10.1109/EMBC48229.2022.9871793 36085909 PMC7613668

[B25] Lee H.-S. Eom K. Park M. Ku S.-B. Lee K. Kim T.. (2023). A multi-channel neural recording system with neural spike scan and adaptive electrode selection for high-density neural interface. IEEE Trans. Circuits Syst. I: Regul. Pap. 70, 2844–2857. 10.1109/TCSI.2023.326868633018948

[B26] Lim J. Moon E. Barrow M. Nason S. R. Patel P. R. Patil P. G.. (2020). “26.9 A 0.19 × 0.17mm2 wireless neural recording IC for motor prediction with near-infrared-based power and data telemetry,” in 2020 IEEE International Solid- State Circuits Conference - (*ISSCC)* (San Francisco, CA: IEEE), 416–418. 10.1109/ISSCC19947.2020.9063005 PMC8919679 35291209

[B27] Liu X. Zhang M. Richardson A. G. Lucas T. H. Van der Spiegel J. (2017). Design of a closed-loop, bidirectional brain machine interface system with energy efficient neural feature extraction and PID control. IEEE Trans. Biomed. Circuits Syst. 11, 729–742. 10.1109/TBCAS.2016.2622738 28029630

[B28] Lorach H. Galvez A. Spagnolo V. Martel F. Karakas S. Intering N.. (2023). Walking naturally after spinal cord injury using a brain–spine interface. Nature 618, 126–133. 10.1038/s41586-023-06094-5 37225984 PMC10232367

[B29] Ma X. Zheng W. Peng Z. Yang J. (2019). “FPGA-based rapid electroencephalography signal classification system,” in 2019 IEEE 11th International Conference on Advanced Infocomm Technology (ICAIT) (Jinan: IEEE), 223–227. 10.1109/ICAIT.2019.8935935

[B30] Malekzadeh-Arasteh O. Pu H. Lim J. Liu C. Y. Do A. H. Nenadic Z.. (2020). An energy-efficient CMOS dual-mode array architecture for high-density ECoG-based brain-machine interfaces. IEEE Trans. Biomed. Circuits Syst. 14, 332–342. 10.1109/TBCAS.2019.2963302 31902769

[B31] Matsushita K. Hirata M. Suzuki T. Ando H. Yoshida T. Ota Y.. (2018). A fully implantable wireless ECoG 128-channel recording device for human brain–machine interfaces: W-HERBS. Front. Neurosci. 12:511. 10.3389/fnins.2018.00511 30131666 PMC6090147

[B32] Maynard E. M. Nordhausen C. T. Normann R. A. (1997). The utah intracortical electrode array: a recording structure for potential brain-computer interfaces. Electroencephalogr. Clin. Neurophysiol. 102, 228–239. 10.1016/S0013-4694(96)95176-0 9129578

[B33] Mestais C. S. Charvet G. Sauter-Starace F. Foerster M. Ratel D. Benabid A. L.. (2015). WIMAGINE: wireless 64-channel ECoG recording implant for long term clinical applications. IEEE Trans. Neural Syst. Rehabil. Eng. 23, 10–21. 10.1109/TNSRE.2014.2333541 25014960

[B34] Metzger S. L. Liu J. R. Moses D. A. Dougherty M. E. Seaton M. P. Littlejohn K. T.. (2022). Generalizable spelling using a speech neuroprosthesis in an individual with severe limb and vocal paralysis. Nat. Commun. 13:6510. 10.1038/s41467-022-33611-3 36347863 PMC9643551

[B35] Moly A. Aksenov A. Martel F. Aksenova T. (2023). Online adaptive group-wise sparse penalized recursive exponentially weighted n-way partial least square for epidural intracranial BCI. Front. Hum. Neurosci. 17:1075666. 10.3389/fnhum.2023.1075666 36950147 PMC10025377

[B36] Musk E. Neuralink. (2019). An integrated brain-machine interface platform with thousands of channels. J. Med. Internet Res. 21:e16194. 10.2196/16194 31642810 PMC6914248

[B37] Neuralink (2024). PRIME Study Progress Update – *User Experience*. Available online at: https://neuralink.com/blog/prime-study-progress-update-user-experience/

[B38] O'Leary G. Pazhouhandeh M. R. Chang M. Groppe D. Valiante T. A. Verma N.. (2018). “A recursive-memory brain-state classifier with 32-channel track-and-zoom δ2 σ ADCs and charge-balanced programmable waveform neurostimulators,” in 2018 IEEE International Solid-State Circuits Conference - (*ISSCC)* (San Francisco, CA: IEEE), 296–298. 10.1109/ISSCC.2018.8310301

[B39] Opri E. Cernera S. Molina R. Eisinger R. S. Cagle J. N. Almeida L.. (2020). Chronic embedded cortico-thalamic closed-loop deep brain stimulation for the treatment of essential tremor. Sci. Transl. Med. 12:eaay7680. 10.1126/scitranslmed.aay7680 33268512 PMC8182660

[B40] Ortega-Martinez A. Von Lühmann A. Farzam P. Rogers D. Mugler E. M. Boas D. A.. (2022). Multivariate Kalman filter regression of confounding physiological signals for real-time classification of fNIRS data. Neurophotonics 9:025003. 10.1117/1.NPh.9.2.025003 35692628 PMC9174890

[B41] Pierce J. R. (1980). An Introduction to Information Theory: Symbols, Signals and Noise, 2nd, rev. edition. New York, NY: Dover publications.

[B42] Rapoport B. I. Turicchia L. Wattanapanitch W. Davidson T. J. Sarpeshkar R. (2012). Efficient universal computing architectures for decoding neural activity. PLoS ONE 7:e42492. 10.1371/journal.pone.0042492 22984404 PMC3440437

[B43] Reich S. Sporer M. Haas M. Becker J. Schüttler M. Ortmanns M.. (2021). A high-voltage compliance, 32-channel digitally interfaced neuromodulation system on chip. IEEE J. Solid-State Circuits 56, 2476–2487. 10.1109/JSSC.2021.3076510

[B44] Shaeri M. Shin U. Yadav A. Caramellino R. Rainer G. Shoaran M.. (2024). A 2.46-mm^2^ miniaturized brain–machine interface (MiBMI) enabling 31-class brain-to-text decoding. IEEE J. Solid-State Circuits 59, 3566–3579. 10.1109/JSSC.2024.3443254

[B45] Shin U. Ding C. Zhu B. Vyza Y. Trouillet A. Revol E. C. M.. (2022). NeuralTree: a 256-channel 0.227-μJ/class versatile neural activity classification and closed-loop neuromodulation SoC. IEEE J. Solid-State Circuits 57, 3243–3257. 10.1109/JSSC.2022.3204508 36744006 PMC9897226

[B46] Shokoueinejad M. Park D.-W. Jung Y. H. Brodnick S. Novello J. Dingle A.. (2019). Progress in the field of micro-electrocorticography. Micromachines 10:62. 10.3390/mi10010062 30658503 PMC6356841

[B47] Spüler M. Walter A. Ramos-Murguialday A. Naros G. Birbaumer N. Gharabaghi A.. (2014). Decoding of motor intentions from epidural ECoG recordings in severely paralyzed chronic stroke patients. J. Neural Eng. 11:066008. 10.1088/1741-2560/11/6/066008 25358531

[B48] Sridhara S. R. DiRenzo M. Lingam S. Lee S.-J. Blazquez R. Maxey J.. (2011). Microwatt embedded processor platform for medical system-on-chip applications. IEEE J. Solid-State Circuits 46, 721–730. 10.1109/JSSC.2011.2108910

[B49] Stanslaski S. Afshar P. Cong P. Giftakis J. Stypulkowski P. Carlson D.. (2012). Design and validation of a fully implantable, chronic, closed-loop neuromodulation device with concurrent sensing and stimulation. IEEE Trans. Neural Syst. Rehabil. Eng. 20, 410–421. 10.1109/TNSRE.2012.2183617 22275720

[B50] Tam W.-k. Wu T. Zhao Q. Keefer E. Yang Z. (2019). Human motor decoding from neural signals: a review. BMC Biomed. Eng. 1:22. 10.1186/s42490-019-0022-z 32903354 PMC7422484

[B51] Tanzarella S. Iacono M. Donati E. Farina D. Bartolozzi C. (2023). Neuromorphic decoding of spinal motor neuron behaviour during natural hand movements for a new generation of wearable neural interfaces. IEEE Trans. Neural Syst. Rehabil. Eng. 31, 3035–3046. 10.1109/TNSRE.2023.3295658 37450365

[B52] Thenaisie Y. Palmisano C. Canessa A. Keulen B. J. Capetian P. Jiménez M. C.. (2021). Towards adaptive deep brain stimulation: clinical and technical note2s on a novel commercial device for chronic brain sensing. J. Neural Eng. 18:042002. 10.1088/1741-2552/ac1d5b 34388744

[B53] Toosi R. Akhaee M. A. Dehaqani M.-R. A. (2021). An automatic spike sorting algorithm based on adaptive spike detection and a mixture of skew-t distributions. Sci. Rep. 11:13925. 10.1038/s41598-021-93088-w 34230517 PMC8260722

[B54] Tsai C.-W. Jiang R. Zhang L. Zhang M. Wu L. Guo J.. (2023). “SciCNN: a 0-shot-retraining patient-independent epilepsy-tracking SoC,” in 2023 IEEE International Solid-State Circuits Conference (ISSCC) (San Francisco, CA: IEEE), 488–490. 10.1109/ISSCC42615.2023.10067518

[B55] Volkova K. Lebedev M. A. Kaplan A. Ossadtchi A. (2019). Decoding movement from electrocorticographic activity: a review. Front. Neuroinform. 13:74. 10.3389/fninf.2019.00074 31849632 PMC6901702

[B56] Wang P. T. Camacho E. Wang M. Li Y. Shaw S. J. Armacost M.. (2019). A benchtop system to assess the feasibility of a fully independent and implantable brain-machine interface. J. Neural Eng. 16:066043. 10.1088/1741-2552/ab4b0c 31585451 PMC7271898

[B57] Wang P. T. Gandasetiawan K. McCrimmon C. M. Karimi-Bidhendi A. Liu C. Y. Heydari P.. (2016). “Feasibility of an ultra-low power digital signal processor platform as a basis for a fully implantable brain-computer interface system,” in 2016 38th Annual International Conference of the IEEE Engineering in Medicine and Biology Society (EMBC) (Orlando, FL: IEEE), 4491–4494. 10.1109/EMBC.2016.7591725 PMC6508955 28325008

[B58] Won M. Albalawi H. Li X. Thomas D. E. (2014). Low-power hardware implementation of movement decoding for brain computer interface with reduced-resolution discrete cosine transform. Annu. Int. Conf. IEEE. Eng. Med. Biol. Soc. 2014, 1626–1629. 10.1109/EMBC.2014.6943916 25570284

[B59] Wu H. Liu Z. Mei J. Tang J. Xu M. Gao B.. (2024). Memristor chip-enabled adaptive neuromorphic decoder for co-evolutional brain-computer interfaces. 10.21203/rs.3.rs-3966063/v1

[B60] Yao L. Zhu B. Shoaran M. (2022). Fast and accurate decoding of finger movements from ECoG through Riemannian features and modern machine learning techniques. J. Neural Eng. 19:016037. 10.1088/1741-2552/ac4ed1 35078156

[B61] Yoo J. Yan L. El-Damak D. Altaf M. A. B. Shoeb A. H. Chandrakasan A. P.. (2013). An 8-channel scalable EEG acquisition SoC with patient-specific seizure classification and recording processor. IEEE J. Solid-State Circuits 48, 214–228. 10.1109/JSSC.2012.2221220

[B62] Yoon D.-Y. Pinto S. Chung S. Merolla P. Koh T.-W. Seo D.. (2021). “A 1024-channel simultaneous recording neural SoC with stimulation and real-time spike detection,” in 2021 Symposium on VLSI Circuits (Kyoto: IEEe), 1–2. 10.23919/VLSICircuits52068.2021.9492480

[B63] Younessi Heravi M. A. Maghooli K. Nowshiravan Rahatabad F. Rezaee R. (2023). A new nonlinear autoregressive exogenous (NARX)-based intra-spinal stimulation approach to decode brain electrical activity for restoration of leg movement in spinally-injured rabbits. Basic Clin. Neurosci. 14, 43–56. 10.32598/bcn.2022.1840.1 37346873 PMC10279987

[B64] Zhang F. Mishra A. Richardson A. G. Otis B. (2011). A low-power ECoG/EEG processing IC With integrated multiband energy extractor. IEEE Trans. Circuits Syst. I: Regul. Pap. 58, 2069–2082. 10.1109/TCSI.2011.2163972

[B65] Zhang Z. Constandinou T. G. (2023). Firing-rate-modulated spike detection and neural decoding co-design. J. Neural Eng. 20:036003. 10.1088/1741-2552/accece 37080210

[B66] Zhong Z. Wei Y. Go L. C. Gu J. (2024). “33.2 A Sub-1μJ/class headset-integrated mind imagery and control SoC for VR/MR applications with teacher-student CNN and general-purpose instruction set architecture,” in 2024 IEEE International Solid-State Circuits Conference (ISSCC), Volume 67 (San Francisco, CA: IEEE), 544–546. 10.1109/ISSCC49657.2024.10454317

